# Quinazolin-4-one Coupled with Pyrrolidin-2-iminium Alkaloids from Marine-Derived Fungus *Penicillium aurantiogriseum*

**DOI:** 10.3390/md10061297

**Published:** 2012-06-07

**Authors:** Fuhang Song, Biao Ren, Ke Yu, Caixia Chen, Hui Guo, Na Yang, Hong Gao, Xueting Liu, Mei Liu, Yaojun Tong, Huanqin Dai, Hua Bai, Jidong Wang, Lixin Zhang

**Affiliations:** 1 CAS Key Laboratory of Pathogenic Microbiology and Immunology, Institute of Microbiology, Chinese Academy of Sciences, Beijing 100101, China; Email: songfuhang@gmail.com (F.S.); phoenix.renbiao@gmail.com (B.R.); chaosyui@gmail.com (K.Y.); chencaixia96@163.com (C.C.); guohui1986@gmail.com (H.G.); angnay@yahoo.cn (N.Y.); gaohong221@gmail.com (H.G.); liuxueting_cn@hotmail.com (X.L.); liumeizky@gmail.com (M.L.); yaojun.tong@gmail.com (Y.T.); huanqindai@gmail.com (H.D.); 2 Graduate University of Chinese Academy of Sciences, Beijing 100049, China; 3 Hisun Pharmaceutical Co. Ltd., Taizhou 318000, China; Email: excitew@yahoo.com.cn (H.B.); jdwang@hisunpharm.com (J.W.)

**Keywords:** marine-derived fungus, *Penicillium aurantiogriseum*, quinazolin-4-one, antitumor

## Abstract

Three new alkaloids, including auranomides A and B (**1** and **2**), a new scaffold containing quinazolin-4-one substituted with a pyrrolidin-2-iminium moiety, and auranomide C (**3**), as well as two known metabolites auranthine (**4**) and aurantiomides C (**5**) were isolated from the marine-derived fungus *Penicillium aurantiogriseum*. The chemical structures of compounds **1**–**3** were elucidated by extensive spectroscopic methods, including IR, HRESIMS and 2D NMR spectroscopic analysis. The absolute configurations of compounds **1**–**3** were suggested from the perspective of a plausible biosynthesis pathway. Compounds **1**–**3** were subjected to antitumor and antimicrobial screening models. Auranomides A–C exhibited moderate cytotoxic activity against human tumor cells. Auranomides B was the most potent among them with an IC_50_ value of 0.097 μmol/mL against HEPG2 cells.

## 1. Introduction

Quinazolin-4-one alkaloids are a class of natural scaffold which has been proved as a drug-like template in medicinal chemistry and considered a favored structure [1]. The quinazolin-4-one ring system has been consistently recognized as a promising pharmacophore because of its broad spectrum pharmacological activities such as antitumor [2], antitubercular [3], anti-HIV [4], anti-inﬂammatory [5], antiangiotensin [6], antibacterial [7], and antifungal [8]. Involved in centrosome separation and bipolar mitotic spindle formation, kinesin spindle protein (KSP) plays an important role in cell division [9]. Some quinazolin-4-one compounds are KSP inhibitors. They can arrest cells in mitosis and induce cell death [10], and have proved to be promising candidates for anticancer drugs [11]. The biological and pharmacological activities of quinazolin-4-one derived compounds have been documented not only from synthetic derivatives but also from several naturally occurring alkaloids isolated from families of the plant kingdom, and from microbes such as *Streptomycetes* and fungi [12,13,14,15,16,17]. 

**Figure 1:**
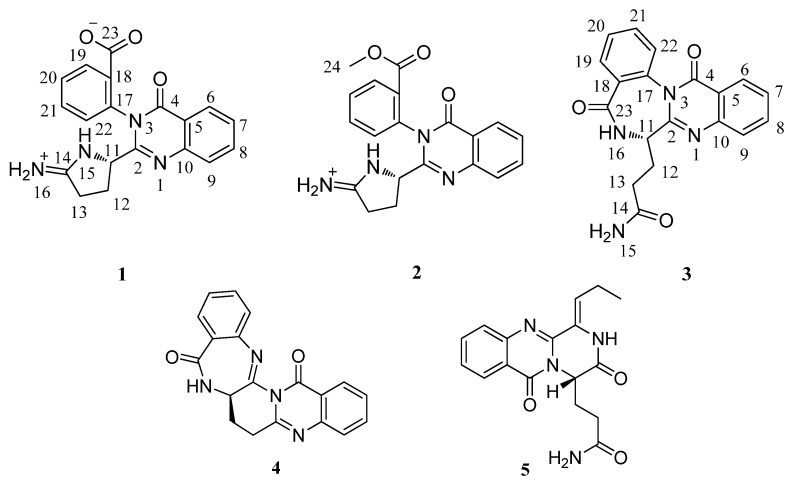
Structures of compounds **1–5**.

During high throughput screening of novel compounds from marine derived microorganisms [18], our group have had identified two pyrone-type polyketides from the marine derived fungus *Penicillium aurantiogriseum* [19]. By varying the culture media according to the OSMAC (one strain-many compounds) approach [20,21], a drastically altered metabolite profile of the same strain was obtained. With the help of HPLC, we found the UV spectra of some compounds in the crude extract were similar to the specific absorbance of the quinazoline-4-one core. Using UV-guided fractionation, three new quinazolin-4-one derivatives (**1**−**3**), together with two known metabolites auranthine (**4**) [22] and aurantiomides C (**5**) [23] (Figure 1) were isolated from this marine-derived fungus. The novel quinazolinone derivatives were named as auranomides A, B and C (**1−3**). Herein, we report the isolation, structure elucidation and bioactivity evaluation of these alkaloids. 

## 2. Results and Discussion

**Figure 2:**
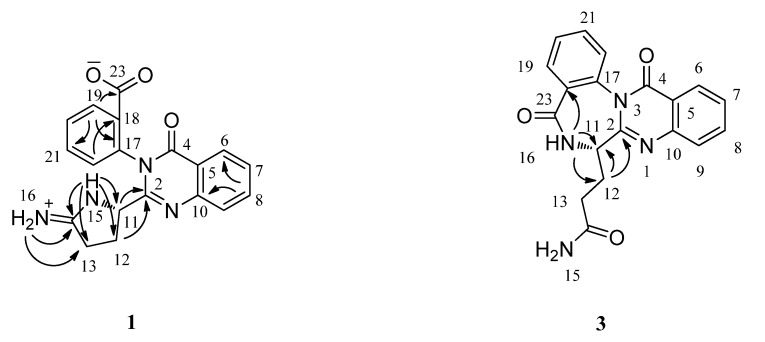
Key HMBC correlations for compounds **1** and **3**.

Compound **1** was obtained as a white amorphous powder with the specific absorbance for the quinazoline-4-one. The molecular formula of **1** was determined to be C_19_H_16_N_4_O_3_ (fourteen degrees of unsaturation) by analysis of its HRESIMS (*m*/*z* 349.1290 [M + H]^+^). The UV spectrum of **1** showed a specific absorbance for the quinazoline-4-one at 216.0, 259.0 and 296.0 nm. The ^1^H, ^13^C, and HSQC spectra of **1** (Table 1), showed 19 carbon signals for two methylene group, one sp^3^ hybrid methine group, eight aromatic methine carbons, five sp^2^ hybrid aromatic quaternary carbons, and three sp^2^ hybrid quaternary carbons at δ_C_ 161.3 (C-4), 165.7 (C-23) and 171.6 (C-14), suggesting the presence of an amide carbonyl, a carboxylic acid, and a C=N carbon, respectively. The ^1^H and ^13^C NMR spectra revealed the presence of an *ortho*-disubstituted benzene ring corresponding to the anthranilate moieties for **1**, which is a fragment of the quinazoline-4-one moiety. Analysis of the ^1^H–^1^H COSY NMR data led to the identification of the fragment from C-11 through C-12 to C-13. In the HMBC spectrum (Figure 2), correlations from H-15 to C-11, C-12, C-13 and C-14, together with correlations from H_2_-N-16 to C-13 and C-14 revealed the pyrrolidin-2-iminium moiety. The HMBC correlations from H-11 and H_2_-12 to C-2 indicated that the pyrrolidin-2-iminium moiety was attached to C-2 through C-11. Protons and carbons signals for another *ortho*-disubstituted benzene ring were observed from the NMR data. Considering the molecular formula, as well as the unsaturation requirements for **1**, this *ortho*-disubstituted benzene ring was attached to N-3 through C-17. In the ^1^H spectrum, three exchangeable protons were observed. The proton at δ_H_ 10.29 was assigned to N-15 by its HMBC correlations to C-11, C-12, C-13 and C-14. The HMBC correlations from the other two exchangeable protons at δ_H_ 9.25 and 8.75 to C-13 and C-14 indicated the carbon signal (δ_C_ 171.6) should be an iminium positive ion. On the basis of these data, the structure of compound **1** was established. 

**Table 1:** NMR spectroscopic data forauranomides A and B (**1** and **2**) in DMSO-*d_6_*.

	Auranomide A				Auranomide B	
Position	δ_C_ mult.	δ_H_ (*J* in Hz)	HMBC ^a^		δ_C_ mult.	δ_H_ (*J* in Hz)
2	155.4, C				155.3, C	
4	161.3, C				161.3, C	
5	120.9, C				120.8, C	
6	126.5, CH	8.12, dd (8.0, 1.0)	4, 8, 10		126.5, CH	8.12, dd (8.0, 1.0)
7	127.2, CH	7.58, t (8.0)	5, 9		127.3, CH	7.59, t (8.0)
8	134.9, CH	7.89, td (8.0, 1.0)	6, 10		135.0, CH	7.90, td (8.0, 1.0)
9	127.1, CH	7.72, d (8.0)	5, 7		127.2, CH	7.73, d (8.0)
10	146.7, C				146.6, C	
11	60.3, CH	4.52, dd (8.5, 5.0)	2, 14		60.2, CH	4.51, dd (8.5, 5.0)
12	27.0, CH_2_	1.98, m	2, 11, 13, 14		26.9, CH_2_	2.00, m
		2.30, m				2.31, m
13	29.3, CH_2_	2.76, m	11, 12, 14		29.3, CH_2_	2.81, 2.75, m
14	171.6, C				171.7, C	
15		N-H, br s, 10.29	11, 12, 13, 14			N-H, br s, 10.32
16		N-H, br s, 8.75	13, 14			N-H, br s, 8.90
		N-H, br s, 9.25	13, 14			N-H, br s, 9.29
17	135.7, C				135.9, C	
18	128.8, C				127.4, C	
19	131.9, CH	8.16, dd (7.5, 1.0)	17, 21, 23		131.8, CH	8.19, dd (7.5, 1.0)
20	130.3, CH	7.72, t (7.5)	18, 22		130.4, CH	7.76, td (7.5, 1.0)
21	133.7, CH	7.84, td (7.5, 1.0)	17, 19		134.3, CH	7.89, td (7.5, 1.0)
22	131.3, CH	7.65, d (7.5)	18, 20		131.5, CH	7.71, d (7.5)
23	165.7, C				164.5, C	
24					52.5, CH_3_	3.66, s

^a^ HMBC correlations, optimized for 8 Hz, are from proton(s) stated to the indicated carbon.

High resolution ESIMS(+) analysis of **2** revealed a pseudomolecular ion at *m/z* 363.1451 [M]^+^, consistent with the molecular formula C_20_H_18_N_4_O_3_, and corresponding to fourteen degrees of unsaturation. Compound **2** had a very similar UV spectrum to that of **1**. The ^1^H, ^13^C, and HSQC spectra of **2** showed 20 carbon signals which were very similar to those of **1** except for the presence of methoxyl group signals (δ_H_ 3.36, δ_C_ 52.5). In the HMBC spectrum, correlation from H_3_-24 to C-23 indicated that the methoxyl group was attached to the carboxyl and generated a carboxylic acid methyl ester. The exchangeable proton at δ_H_ 10.32 was assigned to H-N-15 by the HMBC correlations to C-11, C-12, C-13 and C-14. The other exchangeable protons at δ_H_ 8.89 and 9.24 were assigned to H-N-15 by the HMBC correlations of H-N-16 to C-13 and C-14. On the basis of these data, the structure of compound **2** was established as the methyl ester of **1**.

The molecular formula of **3** was determined to be C_19_H_16_N_4_O_3_ (fourteen degrees of unsaturation) by analysis of its HRESIMS (*m*/*z* 371.1132 [M + Na]^+^) and NMR data (Table 2), the same as that of auranomide A (**1**). It also showed the specific UV spectrum (λ_max_ 219, 259 and 301 nm) for quinazolin-4-one. The ^1^H, ^13^C, and HSQC spectra of **3** showed 19 carbon signals for two methylene groups, one sp^3^ methine group, eight aromatic methine carbons, five sp^2^ aromatic quaternary carbons, and three sp^2^ quaternary carbons at δ_C_ 161.0 (C-4), 166.9 (C-23) and 173.7 (C-14), suggesting the presence of three carbonyl carbons. The ^1^H and ^13^C NMR spectra revealed the presence of an *ortho*-disubstituted benzene ring corresponding to two anthranilate moieties in compound **3**. Analysis of the ^1^H–^1^H COSY NMR data led to the identification of the fragment from C-11 through C-12 to C-13. In the HMBC spectrum (Figure 2), correlations from H-N-16 to C-11, C-12 and C-18, as well as from H_2_-12 to C-2 and C-11 revealed the connection from C-2 through C-11, N-16 to C-23. On the basis of these data, the structure of compound **3** was established.

**Table 2:** NMR spectroscopic data forauranomide C (**3**) in DMSO-*d_6_*.

Position		δ_H_ (*J* in Hz)	HMBC ^a^
2	155.8, C		
4	161.0, C		
5	121.0, C		
6	126.8, CH	8.19, dd (8.0, 1.0)	4, 8, 10
7	128.8, CH	7.60, t (8.0)	5, 9
8	135.2, CH	7.91, td (8.0, 1.0)	6, 10
9	127.4, CH	7.76, d (8.0)	5, 7
10	145.9, C		
11	53.2, CH	4.16, m	2, 12, 13
12	24.1, CH_2_	2.15, 2.34, m	2, 11, 13, 14
13	30.8, CH_2_	2.29, m	11, 12, 14
14	173.7, C		
15		N-H, 6.76, brs	13, 14
		N-H, 7.26, brs	14
16		N-H, 8.82, d, (7.0)	11, 12, 18
17	130.7, C		
18	131.2, C		
19	128.9, CH	7.78, dd (8.5, 1.5)	17, 21, 23
20	127.6, CH	7.59, td (8.5, 1.5)	18, 22
21	128.6, CH	7.67, td (8.5, 1.5)	17, 19
22	133.0, CH	7.64, dd (8.5, 1.5)	18, 20
23	166.9, C		

^a^ HMBC correlations, optimized for 8 Hz, are from proton(s) stated to the indicated carbon.

**Scheme 1:**
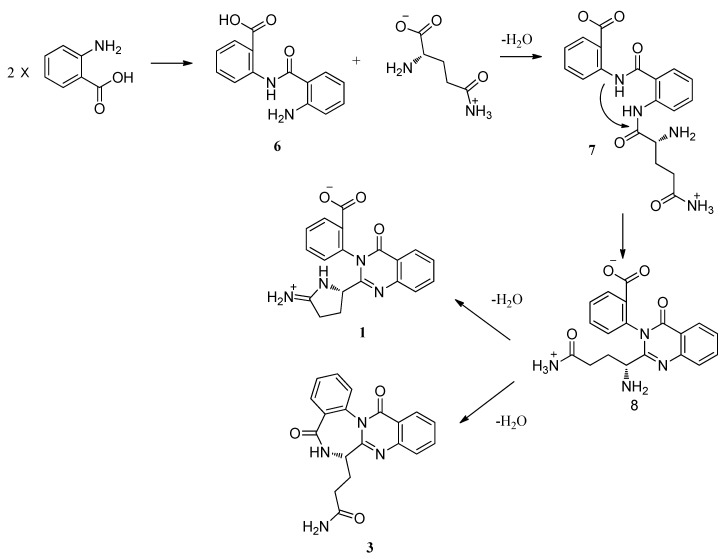
Plausible biosynthesis pathway for quinozolin-4-ones analogues.

Auranomides A and B are a new class of alkaloids which contain the moiety of 2-pyrrolidin-2-iminium quinozoline-4-one. Quinozolin-4-one derivatives have been isolated from several fungi and originated via similar biosynthesis pathways [17,22,23]. Auranthine (**4**) was biosynthesized by *Penicillium aurantiogriseum* [24,25]. Scheme 1 shows a plausible biosynthesis pathway for quinozolin-4-ones analogues (**1**–**3**). Two molecules of anthranilic acid were incorporated into 2-(2-aminobenzamido)-benzoic acid (**6**). A subsequent incorporation of glutamine yielded **7**. The amino group of anthranilic acid reacted with the carbonyl carbons of glutamine to yield **8**. The primary amino group of glutamine could then react with the terminal amide and the carbonyl carbons of anthranilic acid to form **1** and **3**, respectively. From the perspective of biosynthesis, the absolute configurations of C-11 for **1**–**3** were assigned as 11*S*.

Antitumor activity of auranomides A–C (**1**–**3**) was evaluated in several cells lines by the CCK-8 method. As shown in Table 3, auranomide B exhibited the most potent inhibitory effect against human myelogenous leukemia HEPG2 cells, with an IC_50_ value of 0.097 μmol/mL. These compounds were also assessed for activities against methicillin-resistant *Staphylococcus aureus* (MRSA, Clinical isolates, Beijing Chao-yang Hospital, Beijing, China),* Candida albicans* and synergistic antifungal activity with ketoconazole. None of them showed activities at low concentration (MICs > 100 μg/mL). 

**Table 3:** Inhibitory effect of auranomides A–C on the proliferation of tumor cell lines assayed by the CCK-8 method.

Compound	Antitumor (Inhibition Rate at 100 μg/mL)			
	K562	ACHN	HEPG2	A549
Auranomide A	20.48	16.45	16.68	1.04
Auranomide B	76.36	75.31	73.28	30.46
Auranomide C	5.78	8.74	10.72	16.90

## 3. Experimental Section

### 3.1. General Experimental Procedures

UV data were recorded on a Mariner System 5304 instrument. IR spectra were recorded on a Nicolet 5700 FT-IR Microscope spectrometer (FT-IR Microscope Transmission). NMR spectra were recorded on a Varian Inova 500 MHz spectrometer at 500.103 MHz for ^1^H and 125.762 MHz for ^13^C in DMSO-*d*_6_ using solvent signals (DMSO; δ_H_ 2.50/δ_C_ 39.5) as reference; the coupling constants were in Hz. ESIMS spectra were recorded with a ABI Mariner ESI-TOF. Column chromatography was performed with silica gel (200–300 mesh, Qingdao Haiyang Chemical Factory) and Sephadex LH-20 (Pharmacia Co.) columns. HPLC was performed using an Agilent Chromatorex C18 (5 μm) semipreparative column (9.4 × 250 mm). ODs were read by Envision 2103 multilabel reader (PerkinElmer, Waltham, Massachusetts, USA).

### 3.2. Fungal Material and Cultivation

The fungus *Penicillium aurantiogriseum* was obtained from marine mud of the Bohai Sea and identified by analysis of internal transcribed spacer (ITS) regions including ITS1, 5.8S rRNA and ITS2 (GenBank Accession Number: HM587449) and morphology. The strain was deposited at the China General Microbiological Culture Collection Center (CGMCC) in the Institute of Microbiology, Chinese Academy of Sciences, Beijing. The fermentation medium of the strain consisted of 200 g potato infusion, 20 g glucose, 0.25 g (NH_4_)_2_HPO_4_ and 20 g agar powder in 1 L artificial sea water. Altogether, thirty 1 L Erlenmeyer flasks containing 200 mL of the fermentation medium were incubated without rotation at 25 °C for 14 days.

### 3.3. Extraction and Isolation

The fermentation product was exhaustively extracted with EtOAc:MeOH:AcOH (80:15:5) to yield an extract (3.4 g). The residue was suspended in H_2_O and then partitioned with EtOAc. The EtOAc fraction was chromatographed on a reduced pressure silica gel column using a gradient of CH_2_Cl_2_ in MeOH to afford 10 fractions. The third fraction was subjected to a Sephadex LH-20 column [petroleum ether-CH_2_Cl_2_-MeOH (5:5:1)] to give compound **4** (6.5 mg) and four sub-fractions. The third sub-fraction was further purified by reversed-phase HPLC to afford auranomides A (**1**, 1.2 mg), B (**2**, 1.3 mg) and C (**3**, 2.1 mg). The eighth fraction was subjected to a Sephadex LH-20 column [petroleum ether-CH_2_Cl_2_-MeOH (5:5:1)] to give five sub-fractions. The second sub-fraction was further purified by reversed-phase HPLC to afford compound **5** (7.2 mg).

Auranomide A (**1**): White amorphous powder; [α]^20^_D_ +14.9 (*c* 0.10, MeOH); UV (MeOH) λ_max_ (log ε) 296 (3.22), 259 (3.40), 216 (3.93), 213 (3.95) nm; IR ν_max_ 3394, 3189, 1681, 1609, 1473, 1431, 1401, 1275, 1205, 1136, 1049, 1027, 1007 cm^−1^; ^1^H and ^13^C NMR data, see Table 1; ESIMS *m/z* 349 [M + H]^+^; HRESIMS *m/z* 349.1290 [M + H]^+^ (calcd for C_1__9_H_17_N_4_O_3_, 349.1295). 

Auranomide B (**2**): Yellow oil; [α]^20^_D_ +10.8 (*c* 0.10, MeOH); UV (MeOH) λ_max_ 296 (3.28), 259 (3.61), 217 (4.26), 213 (4.26) nm; IR ν_max_ 3194, 2851, 1682, 1609, 1574, 1473, 1436, 1296, 1277, 1203, 1136, 1049, 1027, 1007, 964 cm^−1^; ^1^H and ^13^C NMR data, see Table 1; ESIMS *m/z* 363 [M]^+^; HRESIMS *m/z* 363.1451 [M]^+^ (calcd for C_20_H_19_N_4_O_3_, 363.1452).

Auranomide C (**3**): Yellow oil; [α]^20^_D_ −63.0 (*c* 0.10, MeOH); UV (MeOH); λ_max_ (log ε) 301 (2.72), 259 (3.10), 219 (3.69), 213 (3.68) nm; IR ν_max_ 3404, 2958, 1679, 1619, 1456, 1439, 1392, 1335, 1302, 1257, 1207, 1137, 1028, 1006 cm^−1^; ^1^H and ^13^C NMR data, see Table 2; ESIMS *m/z* 371 [M + Na]^+^; HRESIMS *m/z* 371.1132 [M + Na]^+^ (calcd for C_19_H_16_N_4_O_3_Na, 371.1120).

### 3.4. Antitumor Activity

Human myelogenous leukemia cell line K562, human renal cell carcinoma cell line ACHN, human hepatocellular liver carcinoma cell line HEPG2 and human lung adenocarcinoma cell line A549 were routinely cultured in DMEM supplemented with 10% heat-inactivated fetal bovine serum at 37 °C for 4 h, in an incubator with a humidified atmosphere of 5% CO_2_. The adherent cells at their logarithmic growth stage were digested and were inoculated onto 96-well culture plates at a density of 1.0 × 10^4^ cell/well for the determination of proliferation. Test samples were added to the medium, and incubation was continued for 72 h. Coloration substrate, cell counting kit-8 (CCK-8), was added to the medium followed by further incubation for 3 h. Absorbance at 450 nm with a 600 nm reference was measured thereafter. Medium and DMSO control wells, in which the compound was absent, were included in all of the experiments in order to eliminate the influence of DMSO. The inhibitory rate of cell proliferation was calculated by the following formula (Equation 1):

Inhibition Rate (%) = (OD_control_ − OD_treat_)/OD_control _     (1)

The IC_50_ values (the concentration of a compound that is required for 50% inhibition) were calculated from the corresponding log-dose inhibition curve by the LOGIT method.

### 3.5. Antibacterial Assay

The clinical methicillin-resistant *Staphylococcus aureus* (MRSA) strain was used as the test strain for antibacterial bioassay. Fresh Mueller-Hinton Broth medium (40 μL) was added to each well of a sterilized 96-well microtiter plate (Greiner, Germany), 2 μL of the samples to be tested were added to the test wells, then 40 μL of the test strain solutions were added to each well. The plate was incubated at 37 °C overnight. The anti-MRSA positive control was vancomycin and the minimal inhibition concentrations (MICs) were checked by measuring and comparing the optical densities of the blank control and tested wells. All the experiments were tested in triplicate.

### 3.6. Antifungal and Synergistic Antifungal Assay

*Candida albicans* SC5314 was used as a test strain for the antifungal and synergistic antifungal bioassay according to a previous paper [18]. All the experiments were carried out in flat bottom, 96-well microtiter plates (Greiner, Germany), using a broth microdilution protocol modified from the Clinical and Laboratory Standards Institute M-27A methods [26]. Overnight cultures were picked to prepare the strain solution with medium RPMI 1640 at a concentration of 1 × 10^4^ cfu/mL. Two μL of the samples to be tested was added to the test wells in 96 well-plates followed by 80 μL strain solution. The test plates were incubated at 35 °C for 16 h. The antifungal positive control was ketoconazole and MICs were determined by measuring and comparing the optical densities of the blank control and tested wells. For the synergistic antifungal assay, ¼ of the concentration of ketoconazole needed for inhibition in the antifungal assay was supplemented into the strain solution. All other procedures were the same as the antifungal assay. All the experiments were tested in triplicate.

## 4. Conclusions

By changing the fermentation conditions, such as changing the carbon and nitrogen source as well as the marine derived fungus *Penicillium aurantiogriseum*, completely different natural products were produced including three novel quinazolin-4-ones auranomides A–C. Among them, auranomide B showed moderate antitumor activity. The possible biosynthetic pathway for these novel alkaloids was proposed which begins with two molecules of anthranilic acid and involves one molecule of glutamine. This study proved that the OSMAC approach seems effective in searching for natural products with new structures.
